# Disregarding ZooBank registration results in the unavailability of *Hemicaloosia graminis* Zeng et al., 2012 (Nematoda, Tylenchida) under the ICZN Code

**DOI:** 10.3897/zookeys.309.5532

**Published:** 2013-06-14

**Authors:** R. N. Inserra, J. D. Stanley, A. Troccoli, J. Chitambar, S. A. Subbotin

**Affiliations:** 1 Florida Department of Agriculture and Consumer Services, DPI, Nematology Section, P.O. Box 147100, Gainesville, FL 32614-7100, USA; 2 CNR, Istituto per la Protezione delle Piante, Via G. Amendola 122/D, Bari 70126, Italy; 3 Plant Pest Diagnostic Center, California Department of Food and Agriculture, 3294 Meadowview Road, Sacramento, CA 95832, USA

In September 2012, an Amendment of Articles 8, 9, 10, 21 and 78 of the International Code of Zoological Nomenclature (ICZN) was published in order to expand and refine methods of publication allowed by the Code, particularly in reference to electronic publications (International Commission on Zoological Nomenclature 2012). The amended Article 8.5.3 states that: ”In order for an electronic-published work to be available, it must be registered in the Official Register of Zoological Nomenclature (ZooBank)..... and contain evidence in the work that such registration has occurred.” The requirements for electronic publications also include: clear evidence in the work of the date of publication (Article 8.5.2.) with proof of the occurred registration in ZooBank. The registration in ZooBank is not required for works published in printed journals.

In 2011–2012, R.N. Inserra, J.D. Stanley, A. Troccoli, J. Chitambar and S.A. Subbotin characterized morphologically and molecularly three populations (a total of 42 females and 37 males) of a plant parasitic nematode of the genus *Hemicaloosia* (Hemicycliophoridae) from Florida, and described them as a new species named *Hemicaloosia vagisclera*. The description of this new *Hemicaloosia* was received for publication on 5^th^ March 2012 and published in a preliminary on-line version of *Nematology* on 27^th^ April 2012. This work was not registered in ZooBank because *Nematology* is published as both electronic and printed versions. The paper was published subsequently in a printed version in issue 1 (January) of *Nematology* in 2013 (Inserra et al. 2013). Concomitantly, a team of nematologists including Y. Zeng, W. Ye, L. Tredway, S. Martin and M. Martin characterized morphologically and molecularly two populations (total of 11 females and 1 male) of *Hemicaloosia* from North and South Carolina and described them as a new species named *Hemicaloosia graminis*. The description was received for publication 17^th^ March, 2012 and published only in an on-line version of the issue 2 (June) of *Journal of Nematology* in 2012 (Zeng et al. 2012), without any registration of the work in ZooBank, although *Journal of Nematology* is not a printed Journal.

A morphological comparison of adults of the two *Hemicaloosia* indicates that their morphological features and morphometrics overlap in the two descriptions, in spite of the fact that in the description of *Hemicaloosia graminis* the tail length values for females reported in tables and figures were not in agreement. The two new *Hemicaloosia* also share the same host Bermuda grass (*Cynodon dactylon*). The line drawings for the two species are very similar. A comparison of the morphological features observed at SEM cannot be made because SEM observations are available only for *Hemicaloosia vagisclera* and lack in *Hemicaloosia graminis*. A major characteristic of *Hemicaloosia vagisclera* females consisting of a sclerotized *vagina vera*, from which the name of the Florida species was derived, was not emphasized in the description of *Hemicaloosia graminis*, but well illustrated in line drawings of this species. These results suggest that the two *Hemicaloosia* are morphologically identical. Comparison of 18S rRNA gene sequences for *Hemicaloosia graminis* (JQ446376) and *Hemicaloosia vagisclera* (JQ246425, JQ246426) revealed that in the length of 1496 bp differed in one nucleotide, whereas ITS1 (JQ446376, JQ246427) sequences were identical. The striking morphological and molecular resemblances between these newly described *Hemicaloosia* indicate that they belong to one and the same species.

Taking in account the amended rules of ICZN and the fact that the work by Zeng et al. (2012) was published in electronic form only and no printed version of it was made available, we concluded that the electronic publication by Zeng et al. (2012) with the new species name *Hemicaloosia graminis*, does not meet the requirements of article 8.5.3 of ICZN, as the work itself was not registered in ZooBank. Thus, the name *Hemicaloosia graminis* is not available from the electronically published work of Zeng et al. (2012), whereas the name of *Hemicaloosia vagisclera* is available from the edition printed in January 2013, and is the earliest Code-compliant name for this taxon.
